# Alzheimer's Disease May Benefit from Olive Oil Polyphenols: A Systematic Review on Preclinical Evidence Supporting the Effect of Oleocanthal on Amyloid-β Load

**DOI:** 10.2174/011570159X327650241021115228

**Published:** 2024-10-30

**Authors:** Roberta Zupo, Fabio Castellana, Francesco Panza, Vincenzo Solfrizzi, Madia Lozupone, Roberta Tardugno, Nicola Cicero, Filomena Corbo, Pasquale Crupi, Rodolfo Sardone, Maria Lisa Clodoveo

**Affiliations:** 1 Department of Interdisciplinary Medicine (DIM), University of Bari Aldo Moro, Piazza Giulio Cesare 11, 70100 Bari, Italy;; 2 Department of Interdisciplinary Medicine (DIM), “Cesare Frugoni” Internal and Geriatric Medicine and Memory Unit, University of Bari Aldo Moro, Piazza Giulio Cesare 11, 70100 Bari, Italy;; 3 Department of Translational Biomedicine and Neuroscience “DiBraiN”, University of Bari Aldo Moro, Bari, Italy;; 4 Department of Pharmacy-Drug Sciences, University of Bari “Aldo Moro”, 70125 Bari, Italy;; 5 Department of Biomedical and Dental Sciences and Morphofunctional Imaging, University of Messina, 98168 Messina, Italy;; 6 Department of Agricultural, Food and Forest Science, University of Palermo, Viale delle Scienze, 90128 Palermo, Italy;; 7 Unit of Statistics and Epidemiology, Local Health Authority of Taranto, Taranto, Italy;; 8 Department of Eye and Vision Sciences, University of Liverpool, Liverpool, UK

**Keywords:** Olive oil polyphenols, olecanthal, biophenol, Alzheimer’s disease, amyloid-β, preclinical studies, systematic review

## Abstract

**Background:**

Mediterranean diet may enhance cognitive function and delay the progression of Alzheimer's disease (AD). We conducted a systematic review to investigate the effect of oleocanthal (OC) from extra-virgin olive oil (EVOO) on amyloid-β (Aβ) burden in preclinical models of AD, considering the anti-inflammatory and neuroprotective effects of EVOO biophenols, which are key components of the Mediterranean dietary model.

**Methods:**

The literature was searched through six electronic databases until February 2023. Screening of 52 retrieved articles for inclusion criteria resulted in 7 preclinical reports evaluating the effect of an OC-supplemented diet on AD trajectories by means of Aβ load or clearance in affected models. Reports were appraised for risk of bias using the SYRCLE's RoB tool. A protocol was registered on PROSPERO.

**Results:**

Case control prevailed over the case-crossover design, and the geographical distribution was uniformly American. The study population mostly included 5xFAD, otherwise TgSwDI or wild-type C57BL/6 mouse models. We found a role of OC in reducing Aβ load in the hippocampal parenchyma and microvessels compared with controls. An increased cerebral clearance of Aβ through the blood-brain barrier and a substantial improvement in metabolic and behavioral parameters were also reported in preclinical models under an OC-enriched diet. The risk of bias was shown to be moderate overall.

**Conclusion:**

Preclinical data are promising about the effects of OC from the Mediterranean diet's EVOO in relieving the burden of Aβ in AD; however, further evidence is needed to corroborate the efficacy of this biophenol and strengthen the speculated causal pathway.

## 1 INTRODUCTION

Alzheimer's disease (AD) is the most common form of dementia, affecting about 45 million people worldwide, and is ranked as the fifth leading cause of death globally. It is estimated that more than 5.4 million people have it in the United States [1], while in Europe, dementia affects about 2.5% of people aged 65-69, increasing to about 40 percent of those aged 90-94 [2]. According to the latest data, from an aging population perspective, the number of people with AD will triple by 2050, meaning that 153 million people will be affected by AD globally [2]. Over the past 20 years, most therapeutic approaches have been placed against the production and accumulation of amyloid-β (Aβ) protein outside neurons and the aggregation of tau protein inside cells, widely acknowledged as neuropathological hallmarks of AD [3].

Currently, available drugs for the treatment of AD have only symptomatic effects [3], and there is an unsatisfied need to prevent the onset of AD while delaying or slowing disease progression from mild cognitive impairment (MCI) in the absence of disease-modifying therapies. The substantial success of aducanumab, a monoclonal antibody, in reducing Aβ plaque burden proposed this drug as a potential AD treatment, but the lack of data on overt clinical benefits and the evidence of risks gave rise to some controversy. At present, there are two other different anti-Aβ monoclonal antibodies approved or pending U.S. Food and Drug Administration approval/discontinuation, *i.e*., lecanemab and donanemab. One of the most intriguing and interesting links hypothesized and so far studied is the association between lifestyle factors, such as diet and eating habits, and the onset of AD and dementia [4-7]. Several longitudinal studies have demonstrated the beneficial effect of the Mediterranean Diet (MeDi) model in both halting and slowing the progression of AD [8, 9], as well as improving overall health status [10]. A key component of the MeDi that has been evaluated for its impact on health promotion is extra virgin olive oil (EVOO) [11]. As EVOO is obtained from the first mechanical pressing of the olive fruit [12, 13], it is one of the foods naturally rich in polyphenols, representing the main lipid source in MeDi. A plethora of scientific articles and reviews have so far shown many of the potential health benefits of EVOO to be related to its high content of functional compounds such as polyphenols, tocopherols, carotenoids, sterols, fatty acids and squalene. This specific chemical composition can prevent the incidence of various diseases, such as cardiovascular, neurodegenerative, metabolic, and inflammatory diseases [14-17].

Neurological investigations indicated that EVOO may slow the progression of memory impairment and improve cognitive performance in clinical and preclinical AD models [18-23]. Indeed, supplementing EVOO to the diet of an AD mouse model has been demonstrated to improve the blood-brain barrier (BBB) function, increase the clearance of Aβ, reduce its production, and reduce neuroinflammation [16]. In humans, EVOO biophenols have been associated with neuroprotective activities, mostly attributed to the antioxidant and anti-inflammatory biological pathways. Clinical data from the inCHIANTI study carried out among older adults without dementia, showed that high concentrations of total urinary polyphenols were associated with an approximately 47% lower risk of substantial cognitive decline in global cognitive function and an approximately 48% lower risk of substantial cognitive decline in attention over a 3-year period, but not in executive function [24].

As for the hypothesized causative path, EVOO is well-known to be a source of more than 35 phenolic compounds bearing many antioxidant- and anti-inflammatory features [24, 25]. Yet, while there is evidence that hydroxytyrosol, tyrosol, oleuropein aglycone, and oleacein may have some neuroprotective effect on markers of AD when tested *in vitro* and *in vivo*, poor is known about oleocanthal (OC) [26], another bioactive phenol found in EVOO and known to share similar action to ibuprofen drug for its unique perceptual and anti-inflammatory characteristics [27]. In recent years, OC has become a compound of interest in the search for natural compounds with pharmacological qualities. Following its discovery and identification, it has been reported that oleocanthal has several modes of action in reducing inflammation-related diseases, including neurodegenerative diseases. Here, some evidence has proven OC to be effective against Aβ deposits and other neuropathological features in AD models [19, 28, 29]. Therefore, it is hypothesized that long-term consumption of EVOO-containing OC may contribute to the health benefits associated with the Mediterranean dietary pattern. The present study aimed to systematically review the current preclinical evidence on the effect of the EVOO OC on Aβ load in AD models.

## 2 METHODS

### 2.1 Search Strategy, Study Selection, and Data Extraction

A computerized literature search of MEDLINE and the Cochrane database did not identify any previous systematic reviews on exposure to EVOO OC and Aβ load in AD. However, in light of the lack of clinical studies conducted in humans and given that preclinical studies offer better possibilities for controlled long-term supplementation than clinical studies, the present report considered only studies conducted on cellular and animal models. To conduct this systematic review, we followed the Preferred Reporting Items for Systematic Reviews and Meta-Analyses (PRISMA) criteria, conforming to the 27-item checklist [30]. An a priori protocol for the search strategy and inclusion criteria was established and recorded, with no particular changes to the information provided at registration on PROSPERO, a prospective international registry of systematic reviews (CRD42023512320). We performed separate searches in the U.S. National Library of Medicine (PubMed), Medical Literature Analysis and Retrieval System Online (MEDLINE), EMBASE, Scopus, Ovid, and Google Scholar to find preclinical reports evaluating the effect of OC from EVOO on Aβ load and/or clearance in AD. Therefore, the primary objective was to evaluate whether the administration of this type of biophenol, either meant as OC-enriched EVOO or OC extract supplemented to the standard diet, could have any beneficial effects on AD pathophysiological trajectories by means of estimating the accumulation or clearance of Aβ in affected models. During the title search process, we also considered gray literature using the archive of preprints https://arxiv.org/ in the study selection phase and the database http://www.opengrey.eu/ to access abstracts of notable conferences and other unreviewed material.

No language restriction was used. If necessary, articles in languages other than English were translated by scientists (native speakers of that language) within the University of Bari Aldo Moro. Studies were selected based on title and abstract. When in doubt, the entire publication was purchased and evaluated. Two researchers (RZ, and FC) independently reviewed all abstracts based on the inclusion criteria. Differences were resolved by a third senior scientist (MLC). Studies investigating the effect of OC supplementation in preclinical models of AD with a focus on Aβ deposition were included. However, when they met one of the following exclusion criteria, the articles were excluded: 1) not an original article (*e.g*., review, commentary, letter, opinion, editorial); 2) OC supplementation was combined with other (nutritional) components; 3) an animal model of AD without Aβ pathology was used; 4) absence of a proper control group. As for this latter point, a study was taken as good if the comparator was the negative control (without the intervention).

The search strategy used in PubMed and MEDLINE and adapted to the other four electronic sources included keywords such as “olive oil”, “Alzheimer’s disease”, and “oleocanthal” combined through the use of Boolean indicators such as AND and OR. The search strategy used the Boolean indicator NOT to exclude opinion papers, letters, reviews, and meta-analyses. The literature search had no time restrictions, and papers were retrieved until February 2023. Two researchers (RZ, FC) searched the papers, reviewed the titles and abstracts of the retrieved articles separately and in duplicate, checked the full texts, and selected the articles for inclusion in the study. Inter-rater reliability (IRR) was used to estimate inter-coder agreement and then the κ statistic to measure accuracy and precision. According to PRISMA concepts and the quality assessment steps, a coefficient k of at least 0.9 was obtained in all data extraction steps [31].

### 2.2 Quality Assessment

All reports were critically evaluated independently by two reviewers (RZ, FC) for risk of bias using the SYstematic Review Centre for Laboratory Animal Experimentation risk of bias (SYRCLE's RoB) tool [32]. This tool, which assesses the methodological quality of preclinical studies, features ten items related to six biases, as follows: selection bias, performance bias, detection bias, attrition bias, signaling bias, and other biases. These 10 items include the criteria for evaluating sequence generation, baseline characteristics, allocation concealment, random allocation, blinding (performance bias), random outcome assessment, blinding (detection bias), incomplete outcome data, selective outcome reporting, other sources of bias such as drug pooling, funder influence, risk of project-specific bias, analysis unit errors, and dropout replacement from the original population. Both reviewers assessed each report for risk of bias by coding yes, no, and unclear, indicating low risk, high risk, and not detailed enough to assess the risk of bias, respectively. Finally, any contradictions were reconciled through discussion until a consensus was reached or by consulting a third reviewer (MLC).

## 3 RESULTS

The first systematic search of the literature yielded 52 entries. After excluding duplicates, 20 were classified as potentially relevant and selected for the title and abstract analysis. Then, 13 were excluded for not meeting the characteristics of the approach or the review goal. After reviewing the full text of the remaining records, 7 met the inclusion criteria and were included in the systematic review [19, 21, 29, 33-36]. The Preferred Reporting Items for Systematic Reviews and Meta‐analyses (PRISMA) flow chart illustrating the number of studies at each stage of the review is shown in Fig. (**1**). The final study base included seven articles reporting on the effect of OC on Aβ accumulation in AD models.

Details of the study design, sample size (N), country, author(s) and year of publication, population, intervention type and duration, study setting, outcome measurement method, and major findings are provided in Table **1**. Case-control (5 out of 7, 71.5%) [19, 21, 29, 35, 36] prevailed over case-crossover design (2 out of 7, 28.5%) [19, 33, 34], and the geographical distribution of the studies was uniformly American. The study population included 5xFAD mouse models for 5 of the 7 studies TgSwDI or wild-type C57BL/6. Of these, 5xFAD models are well-known to exhibit Aβ plaque deposition starting at 2 months of age, while gliosis and vascular changes at 3 months. The average duration of the intervention was 3-4 months, except for the older studies by Abuznait and colleagues [19] and Qosa and colleagues [29], which involved a 2- and 4-week intervention. As for the sex, four studies included only males [19, 29, 33, 34], two included only females [21, 35], and one included a mixed male and female population [36]. All animal samples had AD and showed deposits of Aβ at the enrollment time. All studies included a control group and at least one intervention group. As for the intervention, it was either OC-rich EVOO added to a standard diet, or OC extract administered intraperitoneally. Regarding the outcome measurement method, all studies consistently evaluated Aβ levels detected by 6E10 antibodies in brain sections, otherwise, Aβ_1-40_ and Aβ_1-42_ levels in brain tissue lysates and plasma. Of note, a single study, the oldest of all those selected, included an *in vitro* other than *in vivo* analysis [19].

As a main finding, all studies demonstrated a protective potential of OC, both meant as an extract or highly OC-concentrated EVOO, on the clearance and reduction of Aβ deposits in AD preclinical models. Here, specific findings are discussed as follows: Abuznait and colleagues reported results from *in vitro* and *in vivo* data [19]. *In vitro*, they studied the effect of treating bEnd3 cells with vehicle (control) or OC in the presence or absence of inhibitors on the intracellular accumulation of radiolabeled Aβ_1-40_. *In vivo*, however, the mouse in the control group received intraperitoneal vehicle (normal saline) twice daily, while the mouse in the OC group received OC intraperitoneally at a dose of 10 mg/kg twice daily. The results demonstrated a similar and consistent pattern of OC in controlling Aβ levels. In cultured mouse brain endothelial cells, OC treatment had increased the expression and activity of P-glycoprotein (P-gp) and lipoprotein receptor-related protein-1 (LRP1) - major Aβ transport proteins - in brain microvessels, confirming the role of up-regulation of these proteins in enhancing Aβ_1-40_ clearance after OC treatment. In addition, administration of OC extracted from EVOO to wild-type C57BL/6 mice had increased clearance of Aβ_1-40_ from the brain and increased BEI% from 62% in control mice to 79% in OC-treated mice. Qosa and colleagues [29] enrolled a 4-month-old male TgSwDI mouse, demonstrating that 4-week treatment with OC 5 mg/kg/day significantly reduced Aβ burden in hippocampal parenchyma and microvessels compared with controls. This reduction was associated with increased cerebral clearance of Aβ through the BBB. Al Rihani and colleagues [21] also enrolled TgSwDI mice, but female and 6-month-old. Compared with the control group treated with refined olive oil (ROO), the intervention involved the administration of OC-rich EVOO, providing 476 (μg/kg)/day of OC. The results showed that, compared with the control group, OC-rich EVOO oil significantly reduced total Aβ 6E10 detected by 61% and 73% in the cerebral cortex and hippocampus, respectively. The authors provided proof that long-term dietary supplementation with OC-rich EVOO significantly reduced inflammasome activation through inhibition of NLRP3 and increased autophagy through activation of the AMPK-ULK1 pathway compared with mice consuming a diet enriched with refined olive oil.

Batarseh and Kaddoumi [36], Tajmim and colleagues [35], Yang and colleagues [34], and Abdallah and colleagues [33] enrolled 5xFAD mouse models. Of them, Batarseh [36] evaluated three intervention groups, *i.e*., EVOO (476 μg/kg/day oleocanthal), donepezil (1 mg/kg/day), and EVOO plus donepezil (1 mg/kg/day), other than the control group. The results showed that EVOO consumption in combination with donepezil significantly reduced Aβ load and related pathological changes. One possible explanation was the enhancement of the Aβ elimination pathway, including BBB clearance and enzymatic degradation, and the shift of amyloid precursor protein (APP) processing to the non-amyloidogenic pathway. The study by Tajmim and colleagues [35] aimed to develop new oral OC formulations, evaluating whether they maintained OC activity on Aβ pathology attenuation in a 5xFAD mouse model after 4-month oral dosing. To this aim, the powder formulation of OC (OC-PF) and the solid dispersion formulation of OC with erythritol (OC-SD) were prepared and characterized using FT-IR spectroscopy, powder X-ray diffraction, and scanning electron microscope (ScEM) analysis. Both formulations showed an improved dissolution profile of OC. OC-SD and OC-PF treatments attenuated Aβ plaque deposition evidenced by Congo red staining of 5xFAD mouse brain sections. OC-PF treatment significantly reduced the intensity of Aβ deposition compared with vehicle control in both hippocampal and cortex regions. OC-SD significantly reduced Aβ deposition in the hippocampal region compared with the vehicle control group. OC-SD treatment attenuated overall Aβ deposition but not to the level of statistical significance in the cortex region. However, both OC-PF and OC-SD treatment showed modulatory effects on Aβ accumulation in both the hippocampus and cortex of the 5xFAD mouse brain compared with the vehicle control group. Yang and colleagues [34] administered a 10 mg/kg OC intervention on male 4-month- and 9-month-old 5xFAD mice, showing that in the brain, OC significantly reduced Aβ_1-40_ levels by 40% and 73% in 4-month- and 9-month-old mice, respectively, and Aβ_1-42_ levels by 45% in 9-month-old 5xFAD mice. Finally, the recent study by Abdallah and colleagues [33] compared the beneficial effects of equivalent doses of OC-low EVOO (0.5 mg total phenolic content/kg) and OC (0.5 mg OC/kg) on Aβ and related pathology and evaluated their effect on neuroinflammation in a 5xFAD mouse model with advanced AD pathology. Homozygous 5xFAD mice were fed refined olive oil (ROO), low OC EVOO, or OC for 3 months starting at the age of 3 months. Compared with the ROO group, EVOO significantly reduced Aβ_1-42_ by 35%; however, it did not reach significance for its effect on Aβ_1-40_ levels, which decreased by 30% due to high variability in the ROO group. OC, on the other hand, was able to reduce both isoforms by about 50% significantly. While no significant difference was observed between EVOO and OC on reduced Aβ_1-40_ levels, OC demonstrated a significant reduction in Aβ_1-42_ levels compared to EVOO.

**Fig. (2):**
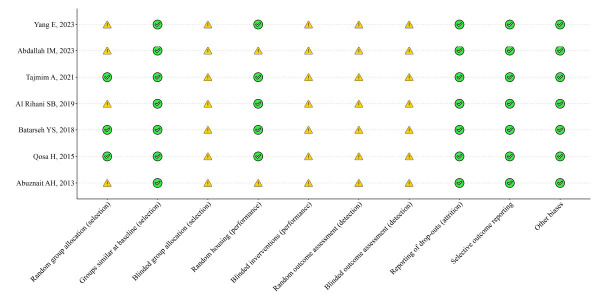
Risk of bias across the 10 domains of SYRCLE's RoB tool.

The risk of bias across the 10 domains of SYRCLE's RoB tool was shown to be moderate overall (Fig. **2**). Specifically, a reduced risk was found for domains 2, 8, 9, and 10 across the selected studies. Domains 2 and 4 raised some concerns for a few studies, while the remaining domains showed unclear risk due to a lack of information.

## 4 DISCUSSION

The aim of this systematic review was to sum up and analyze the current preclinical evidence on the effect of dietary supplementation of OC, a biophenol found in EVOO, on Aβ load in preclinical models of AD. As AD is well-known to exhibit several hallmarks, including Aβ plaques, impaired BBB, diffusely activated glial cells, synaptic dysfunction, neuroinflammation, and neuronal death, this research was planned to focus on Aβ burden as a major, overstudied AD feature. To the best of our knowledge, the present systematic review was the first study to summarize data, including preclinical evidence on the efficacy of this bioactive compound in AD. As a major finding, the results consistently proved the role of OC in reducing Aβ load in the hippocampal parenchyma and microvessels compared with untreated controls (vehicle or ROO). This drop was also associated with increased cerebral clearance of Aβ through the BBB and a substantial improvement in metabolic and behavioral parameters in mouse models treated with an OC-enriched diet.

To discuss these findings, it is to be kept in mind that the MeDi is well-known to exert several beneficial effects on neuronal health and has been associated with slowing cognitive impairment and dementia in large-scale clinical and preclinical studies [7, 37-42]. From a global perspective, leading intervention studies on the MeDi, not neglecting the analysis of the network of microbial ecosystems, have shown that bacterial taxa featuring this diet pattern occupy key interaction positions in the mechanisms that promote healthy neuronal aging [43]. Synergistic interactions between inflammatory, oxidative stress-related, and metabolic mechanisms have been suggested to be responsible for many neuroprotective benefits. Of note, the known abundance of mono- and poly-unsaturated fatty acids (PUFAs), micro- and macronutrient density, and fiber content constitute a cocktail of potent multiple bioprotective compounds that mediate the diet-gut-microbiota-brain interaction. Among the prominent foods, olive oil (and especially EVOO) as the main source of fat in the MeDi is known to exert anti-inflammatory, antioxidant, and neuroprotective effects [11, 12, 14, 15], mainly due to its load of phenolic compounds such as hydroxytyrosol, OC, tyrosol, oleuropein aglycone, oleacein and luteolin.

Deepening the role of biophenols, among the other [28, 44-47], OC has been described as having strong anti-inflammatory activity similar to that of ibuprofen, a nonsteroidal anti-inflammatory drug (FANS), as able to inhibit cyclooxygenase enzymes. Along these lines, this research clearly shows that OC works well in modulating AD physiopathological mechanisms by interfering with the aggregation of peptides and proteins involved in AD pathogenesis, bypassing the rise of toxic species, and promoting the formation of nontoxic aggregates. Of particular interest is the oldest report by Abuznait and colleagues [19], collecting both *in vitro* and *in vivo* data and indicating that OC increases Aβ clearance by regulating P-gp and also LRPI. The authors focused on two peptide species that have been implicated in AD, both Aβ_1-40_ and Aβ_1-42_ in mouse brain endothelial cells. They concluded that OC significantly increased the clearance rate of Aβ_1-40_ in these cells. In addition, Abuznait and colleagues tested OC for the first time *in vivo* (10 mg/kg/day, twice daily for 2 weeks), showing that after OC administration, the clearance rate and the degradation of Aβ_1-40_ were increased. Consistently, other retrieved preclinical data showed that the supplementation of diet with OC for an average period of 3-4 weeks significantly reduces the Aβ load in the hippocampal parenchyma and microvessels [21, 29, 33-36].

As possible underlying paths postulated, OC may act through the following mechanisms: an increase in cerebral clearance of Aβ through the BBB [20, 29, 36] by induction of increased expression of Aβ clearance proteins (P-gp and LRP1) at the level of the BBB and activation of the apolipoprotein E-dependent amyloid clearance pathway in mouse brain [19]; a reduction in Aβ production by shifting APP processing to the non-amyloidogenic pathway [35]; a reduction of neurodegenerative disease markers, as an ibuprofen analog [25]; a reduction of AD-associated pathology by reducing neuroinflammation through inhibition of NACHT inflammasome, LRR and PYD domain-containing protein 3 (NLRP3) and inducing autophagy through activation of the AMP-activated protein kinase (AMPK)/Unc-51-like autophagy activating kinase 1 (ULK1) pathway [21]. In light of these favorable mechanisms, however, it is also worth considering that, unfortunately, one of the main obstacles to the application of OC as a potential nutraceutical could be its irritating, pungent and astringent taste, similar to that of ibuprofen drug.

## 5 LIMITATIONS

There are some limitations to discuss around the biological potential of biophenols. Certainly, there is strong evidence that OC may be an effective anti-inflammatory agent and demonstrated pharmacological-like actions in preclinical studies. However, more preclinical and future clinical studies are needed to fully elucidate the potential of this compound in AD and other neurodegenerative diseases. Extrapolation of *in vitro* to *in vivo* results presents many difficulties, and care must be taken when reporting the effects of a compound removed from the matrix in which it is normally contained. The phenols in EVOO act synergistically and complement each other in terms of anti-inflammatory, antioxidant, and antimicrobial properties, so OC, in addition to the other phenols in EVOO, plays a role in the health benefits associated with EVOO intake. Another limitation may be due to the small amount of data considered for the present systematic review, although it should be considered that this is preclinical evidence and still an emerging topic. Lastly, the authors of the selected studies did not add information on olive oil cultivar and interannual factors involved, thus making the monounsaturated fatty acid profile heterogeneous.

## CONCLUSION

Existing preclinical literature on OC in AD outlined the promise of this biophenol for efficacy in relieving Aβ burden. Furthermore, preclinical and clinical evidence is needed to corroborate the association and potentiate the speculated causative pathway.

## Figures and Tables

**Fig. (1):**
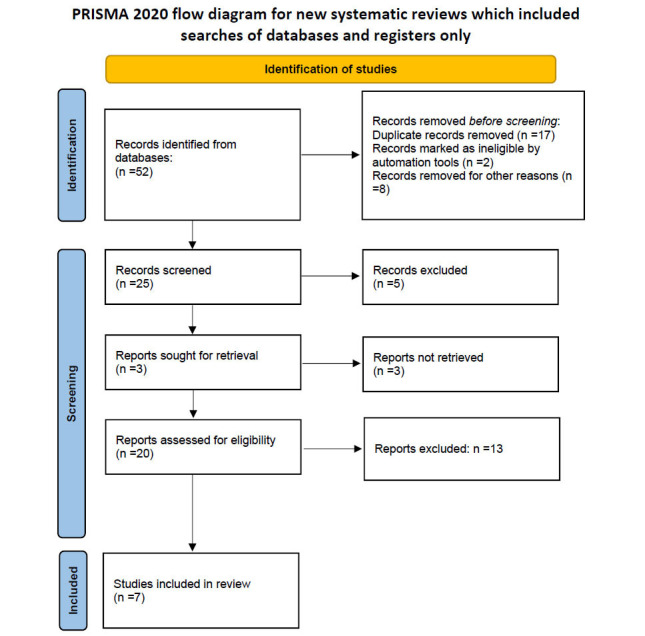
PRISMA flowchart.

**Table 1:** Description of the selected studies N= 7.

**First Author, Year**	**Study Design**	**Outcome**	**N**	**Country**	**Population**	**Intervention**	**Intervention Duration**	**Outcome ** **Measurement Method**	**Major Findings**
Abdallah IM, (2023) [33]	Case crossover	Brain Aβ levels	30	USA	Male 5xFAD mouse, 1.5-month-old	**• **Control (ROO: <10 mg/kg total biophenol) **•** EVOO (total phenolic content of 540 mg/kg, with OC less than 35 mg/kg) **• **OC (ROO + OC at 540 mg/kg)	3-months	Total Aβ levels detected by 6E10 antibodies in brain sections	Compared with the ROO group, EVOO significantly reduced Aβ42 by 35%; however, it did not reach significance for its effect on Aβ40 levels, which decreased by 30% due to high variability in the ROO group; OC, on the other hand, was able to significantly reduce both isoforms by about 50%. While no significant difference was observed between EVOO and OC on reduced Aβ_1-40 _levels, OC demonstrated a significant reduction in Aβ_1-42 _levels compared to EVOO.
Yang E, (2023) [34]	Case crossover	Brain Aβ levels	20	USA	Male 5xFAD mouse, 4-month- and 9-month-old	**• **Control **• **OC 10 mg/kg	3-months	Aβ_1-40_ and Aβ_1-42_ levels in brain tissue lysates and plasma	In the brain, OC significantly reduced Aβ_1-40 _by 40% and 73% in 4- and 9-month-old mouse, respectively, and Aβ_1-42_ levels by 45% in 9-month-old 5xFAD mouse.
Tajmim A, (2021) [35]	Case control	Aβ accumulation in both the hippocampus and cortex regions	18	USA	Female 5xFAD mouse, 4-week-old	**• **Control **• **OC-PF equivalent to OC 10 mg/kg **• **OC-SD equivalent to OC 10 mg/kg	4-months	Total Aβ levels detected by 6E10 antibodies in brain sections	OC-SD and OC-PF treatments attenuated Aβ plaque deposition evidenced by Congo red staining of 5xFAD mouse brain sections. OC-PF treatment significantly reduced the intensity of Aβ deposition compared with vehicle control in both hippocampal and cortex regions. OC-SD significantly reduced Aβ deposition in the hippocampal region compared with the vehicle control group. OC-SD treatment attenuated overall Aβ deposition but not to the level of statistical significance in the cortex region. However, both OC-PF and OC-SD treatment showed modulatory effects on Aβ accumulation in both the hippocampus and cortex regions of the 5xFAD mouse brain compared with the vehicle control group.
Al Rihani SB, (2019) [21]	Case control	BBB function, Aβ-related pathology, and MWM performance	14	USA	Female TgSwDI mouse, 6-month-old	**•** Control (ROO) **• **OC-rich EVOO, providing 476 (μg/kg)/ day of OC	3-months	Total Aβ levels detected by 6E10 antibodies in brain sections	Compared to control group, EVOO significantly reduced total 6E10-detected Aβ by 61% and 73% in brain cortex and hippocampus. Data demonstrated that long-term dietary supplementation with OC-rich EVOO significantly reduced inflammasome activation through NLRP3 inhibition and increased autophagy through AMPK-ULK1 pathway activation when compared to mouse consuming a refined olive oil-enriched diet.
Batarseh YS, (2018) [36]	Case control	Amyloid-β load and related toxicity	12	USA	Male and female 5xFAD mouse, 1-month-old	**•** Control **•** EVOO (476 μg/kg/day oleocanthal) **•** Donepezil (1mg/kg/day) **•** EVOO + donepezil (1mg/kg/day)	3-months	Total Aβ levels detected by 6E10 antibodies in brain sections	Our results showed EVOO consumption in combination with donepezil significantly reduced Aβ load and related pathological changes. Reduced Aβ load could be explained, at least in part, by enhancing Aβ clearance pathways including blood-brain barrier (BBB) clearance and enzymatic degradation and shifting amyloid precursor protein (APP) processing toward the non-amyloidogenic pathway.
Qosa H, (2015) [29]	Case control	Aβ clearance from the brain	12	USA	Male TgSwDI mouse, 4-month-old	**•** Control **•** OC 5 mg/kg/day	4-weeks	Total Aβ levels detected by 6E10 antibodies in brain sections	Mouse treatment for 4 weeks with oleocanthal significantly decreased amyloid load in the hippocampal parenchyma and microvessels. This reduction was associated with enhanced cerebral clearance of Aβ across the BBB.
Abuznait AH, (2013) [19]	Case control	Aβ clearance from the brain *via* up-regulation of P-gp and LDL LRP1, major Aβ transport proteins, at the BBB	8	USA	Male C57BL/6 wild-type mouse, 6-7 weeks old	**• **Control **• **OC 10 mg/kg/day, twice daily	2-weeks	Total Aβ levels detected by 6E10 antibodies in brain sections	The results of *in vitro* and *in vivo* data demonstrated a similar and consistent pattern of oleocanthal in controlling Aβ levels. In brain endothelial cells of cultured mouse, oleocanthal treatment increased the expression and activity of P-gp and LRP1. Data on the BEI% of Aβ_1-40_ showed that administration of oleocanthal extracted from extra virgin olive oil to wild-type C57BL/6 mouse increased the clearance of Aβ_1-40_ from the brain and increased the BEI% from 62% in control mouse to 79% in OC-treated mouse. The increased expression of P-gp and LRP1 in brain microvessels and inhibition studies confirmed the role of up-regulation of these proteins in enhancing the clearance of Aβ_1-40 _after oleocanthal treatment.

**Abbreviations:** OC: Oleocanthal; Aβ (Amyloid-β); ROO (Refined Olive Oil); EVOO (Extra-virgin olive oil); OC-PF (OC powder formulation); OC-SD (OC-solid dispersion formulation with erythritol); P-gp (P-glycoprotein); LRP1 (lipoprotein receptor related protein-1); BBB (blood-brain barrier); MWM (Morris Water Maze Testing); APP (amyloid precursor protein); BEI (brain efflux index).

## Data Availability

All data supporting the findings of this study are available from the corresponding author (MLC) upon reasonable request.

## LIST OF ABBREVIATIONS

AD: Alzheimer's Disease
Aβ: Amyloid-β
BBB: Blood-brain Barrier
EVOO: Extra Virgin Olive Oil
LRP1: Lipoprotein Receptor-related Protein-1
MCI: Mild Cognitive Impairment
ROO: Refined Olive Oil

## STANDARDS OF REPORTING

PRISMA guidelines and methodology were followed.

## SUPPLEMENTARY MATERIAL

PRISMA checklist is available as supplementary material on the publisher’s website along with the published article.
